# Assessing the composition of microbial communities in textile wastewater treatment plants in comparison with municipal wastewater treatment plants

**DOI:** 10.1002/mbo3.413

**Published:** 2016-09-25

**Authors:** Ken Meerbergen, Maarten Van Geel, Michael Waud, Kris A. Willems, Raf Dewil, Jan Van Impe, Lise Appels, Bart Lievens

**Affiliations:** ^1^ Laboratory for Process Microbial Ecology and Bioinspirational Management (PME&BIM)Department of Microbial and Molecular Systems (M^2^S)Technology Campus De NayerKU LeuvenSint‐Katelijne‐WaverBelgium; ^2^ Plant Conservation and Population BiologyDepartment of BiologyKU LeuvenLeuvenBelgium; ^3^ Process and Environmental Technology Lab (PETLab)Department of Chemical EngineeringTechnology Campus De NayerKU LeuvenSint‐Katelijne‐WaverBelgium; ^4^ Chemical and Biochemical Process Technology and Control (BioTeC)Department of Chemical EngineeringTechnology Campus GentKU LeuvenGentBelgium

**Keywords:** 454‐pyrosequencing, activated sludge, environmental fit, microbial community, quantitative real‐time PCR, wastewater

## Abstract

It is assumed that microbial communities involved in the biological treatment of different wastewaters having a different chemical composition harbor different microbial populations which are specifically adapted to the environmental stresses encountered in these systems. Yet, little is known about the composition of these microbial communities. Therefore, the aim of this study was to assess the microbial community composition over two seasons (winter and summer) in activated sludge from well‐operating textile wastewater treatment plants (WWTPs) in comparison with municipal WWTPs, and to explain observed differences by environmental variables. 454‐pyrosequencing generated 160 archaeal and 1645 bacterial species‐level Operational Taxonomic Units (OTUs), with lower observed richness in activated sludge from textile WWTPs compared to municipal WWTPs. The bacterial phyla Planctomycetes, Chloroflexi, Chlorobi, and Acidobacteria were more abundant in activated sludge samples from textile WWTPs, together with archaeal members of Thaumarchaeota. Nonmetric multidimensional scaling analysis of the microbial communities showed that microbial communities from textile and municipal WWTPs were significantly different, with a seasonal effect on archaea. Nitrifying and denitrifying bacteria as well as phosphate‐accumulation bacteria were more abundant in municipal WWTPs, while sulfate‐reducing bacteria were almost only detected in textile WWTPs. Additionally, microbial communities from textile WWTPs were more dissimilar than those of municipal WWTPs, possibly due to a wider diversity in environmental stresses to which microbial communities in textile WWTPs are subjected to. High salinity, high organic loads, and a higher water temperature were important potential variables driving the microbial community composition in textile WWTPs. This study provides a general view on the composition of microbial communities in activated sludge of textile WWTPs, and may provide novel insights for identifying key players performing important functions in the purification of textile wastewaters.

## 1 Introduction

Wastewater treatment using activated sludge processes has been commonly practiced to purify municipal and industrial wastewater, mainly because of the high treatment efficiency and low operating cost. Activated sludge processes are based on the ability of microorganisms to utilize organic material as a source of energy and/or as a source of carbon and other minerals for growth (Carneiro, Umbuzeiro, Oliveira, & Zanoni, 2010), thereby playing important roles in the biodegradation of organic materials, transformation of toxic compounds into harmless products, and removal of nutrients such as ammonia, nitrate, sulfate, and phosphate (Gentile, Jessup, Nyman, & Criddle, 2007; Wang et al., 2011). The stable operation of biological wastewater treatment plants (WWTPs) relies upon the occurrence, relative abundance and activity of several microbial populations in the activated sludge performing these processes (Gentile et al., 2007; Wagner & Loy, 2002; Yang et al., 2014). As variations in microbial community composition are often associated with changes in the functional capabilities of the communities, microbial community and functional stability are generally recognized as important factors to efficiently treat wastewater (Wang, Xia, Wen, Yang, & Zhou, 2014).

Microbial communities of activated sludge in WWTPs have been intensively studied over the last decade, especially for WWTPs dealing with municipal wastewater (e.g., López‐Vázquez, Hooijmans, Brdjanovic, Gijzen, & van Loosdrecht, 2008; Miura et al., 2007; Sanapareddy et al., 2009; Wang et al., 2010, 2011). In general, trends are observed with members of the phylum Proteobacteria, frequently being the most abundant in municipal WWTPs (accounting for 30%–60% of the total number of sequences), followed by Actinobacteria and Bacteroidetes (Hu, Wang, Wen, & Xia, 2012; Ju, Guo, Ye, Xia, & Zhang, 2014; Saunders, Albertsen, Vollertsen, & Nielsen, 2016; Wang, Hu, Xia, Wen, & Ding, 2012; Wei et al., 2015; Ye & Zhang, 2013; Zhang, Shao, & Ye, 2012; Zhao et al., 2014). Moreover, in a recent Illumina MiSeq‐based study of 13 municipal WWTPs across Denmark, it was shown that the plants contained a core community of 63 abundant genus‐level operational taxonomic units (OTUs), indicating that microbial communities of activated sludge in municipal WWTPs are quite similar across multiple plants (Saunders et al., 2016). However, it is reasonable to assume that communities involved in the biological treatment of more hazardous wastewaters, such as those originating from the textile industry, harbor different microbial populations that are specifically adapted to the environmental stresses encountered in these systems. Textile industry effluents typically contain high concentrations of dyes, dyeing additives, and diverse chemicals, some of which are nonbiodegradable, toxic, mutagenic, or carcinogenic, which pose a major threat to health and environment. Additionally, textile wastewater generally has a low biological oxygen demand/chemical oxygen demand (BOD/COD) ratio (around 20%), a wide range of pH (4–12), and may contain several inhibitor compounds (hampering effective biological wastewater treatment), active substances, adsorbable organic halogens (e.g., chlorine compounds) (AOX) and high salt concentrations, altogether making textile wastewater difficult to treat (Sandhya & Swaminathan, 2006; Selcuk, 2005; Verma, Dash, & Bhunia, 2012; Wu, Wang, Kong, Liu, & Xia, 2007). Therefore, it can be hypothesized that microbial communities in textile WWTPs are different from those observed in municipal WWTPs. However, so far only little is known about the microbial community composition and their functioning in activated sludge from textile wastewater treatment systems (but see Yang et al., 2014).

The aim of this study was to assess the microbial community composition in activated sludge from textile WWTPs in comparison with municipal WWTPs in Flanders (Belgium), and to explain observed differences by environmental factors. The first objective of this study was to assess differences in the microbial (both bacterial and archaeal) communities using 454 amplicon pyrosequencing and real‐time quantitative PCR (qPCR). Secondly, we aimed at determining which environmental factors drive species richness, diversity, and community composition in activated sludge from different WWTPs dealing with different wastewaters.

## 2 Materials and Methods

### 2.1 Study samples

Activated sludge samples (0.5 L) were collected in triplicate from aerobic tanks of five textile WWTPs and five municipal WWTPs. Additionally, samples were taken from one plant dealing with both textile and municipal wastewater. All WWTPs were located in Flanders (Belgium) and were characterized by a stable operating system, discharging wastewater effluents within legal standards. Sampling was performed in two different seasons, including winter (February 2015) and summer (July 2015). Following sampling, samples were immediately centrifuged at 3500 g using a portable Hettich EBA 20 centrifuge (Hettich Lab Technology, Tuttlingen, Germany) to precipitate the sludge. Approximately 1 g of precipitated sludge was resuspended in 20 ml RNAlater (Life Technologies, Carlsbad, CA, USA) to preserve the nucleic acids present in the samples. At the same time, influent samples (1 L) were collected from each wastewater for chemical analysis. Samples were transported in an ice‐cooled container to the laboratory and stored overnight at 4°C prior to further analysis.

### 2.2 DNA extraction, PCR amplification, and 454 amplicon pyrosequencing

Following centrifugation of the samples (10 ml), genomic DNA was extracted from 0.15 g precipitated material using the Power Soil DNA isolation kit (MoBio Laboratories Inc., Solana Beach, CA, USA) according to the manufacturer's instructions. Subsequently, DNA extracts from the three samples taken per studied WWTP were pooled and stored at −80°C until further processing.

Amplicon libraries were created using two PCR primer sets, targeting part of the bacterial and archaeal 16S ribosomal RNA (rRNA) genes, including the primer pairs S‐D‐Bact‐0341‐b‐S‐17 / S‐D‐Bact‐0785‐a‐A‐21 (covering the V3‐V4 region; amplicon size of approximately 464 bp; Klindworth et al. 2013) and S‐D‐Arch‐0519‐a‐S‐15 / S‐D‐Arch‐1041‐a‐A‐18 (covering the V4‐V6 region; amplicon size of approximately 540 bp; Klindworth et al. 2013), respectively. Previous research has shown that these primer pairs were successfully implemented for water research and outperformed other primer pairs for classical and next‐generation sequencing‐based diversity studies (Klindworth et al. 2013; Connelly, Baer, Cooper, Bronk, & Wawrik, 2014; Richert et al., 2015; Savio et al., 2015; Shan et al., 2015). “Fusion” primers, required for the 454‐pyrosequencing process, were designed according to the guidelines for 454 GS‐FLX+ Titanium Lib‐L sequencing and contained the Roche 454‐pyrosequencing adapters and a sample‐specific multiplex identifier (MID) sequence in between the adapter and the forward primer for sample‐specific sequence tracking (Table S1).

PCR amplification was performed in duplicate on each pooled DNA sample using a T100 Thermal Cycler (Bio‐Rad, Hercules, CA, USA) in a reaction volume of 20 μl, containing 1.0 μl 10× diluted genomic DNA, 1.5 μl dNTP mixture (2 mmol/L stock; Invitrogen, Carlsbad, CA, USA), 0.5 μl of each primer (20 μmol/L stock), 2.0 μl 10× Titanium Taq PCR buffer, 0.4 μl Titanium Taq DNA polymerase (Clontech Laboratories, Palo Alto, CA, USA), and 14.1 μl nuclease‐free water. PCR conditions were as follows: initial denaturation of 2 min at 94°C, followed by 30 cycles of denaturation at 94°C for 45 s, annealing at 61°C (bacteria) or 63°C (archaea) for 45 s and extension at 72°C for 60 s, and a final extension step for 10 min at 72°C. Following agarose gel electrophoresis, amplicons of the expected size range were excised and extracted from the gel using the QIAquick gel extraction kit, according to the manufacturer's instructions (Qiagen, Hilden, Germany). Purified dsDNA amplicons were quantified using a Qubit 2.0 fluorometer and the high‐sensitivity DNA reagent kit (Invitrogen, Carlsbad, CA, USA), and diluted to equimolar concentrations. Subsequently, quantifications were verified using a qPCR assay on an ABI StepOnePlus real‐time PCR instrument (Life Technologies, Carlsbad, CA, USA) using primers that hybridize to the fusion primer adapter sequences (emPCR_F (forward): ‘5‐CCATCTCATCCCTGCGTGTCTCCGACTCAG‐’3; emPCR_R (reverse): ‘5‐CCTATCCCCTGTGTGCCTTGGCAGTCTCAG‐’3). Reactions were performed in a volume of 20 μl consisting of 10.0 μl of 2× iTaq universal SYBR Green supermix (Bio‐Rad Laboratories, Hercules, CA, USA), 1.0 μl DNA template, 0.5 μl of each primer (20 μmol/L stock), and 8.0 μl nuclease‐free water. Amplifications were run as follows: initial denaturation for 2 min at 95°C, followed by 40 cycles of 15 s denaturation at 95°C, 30 s annealing at 58° and 30 s extension at 60°C. At the end of the qPCR run, a melting curve analysis was performed to confirm product specificity (60–95°C, ΔT per 15 s = 0.3°C). Quantification was performed using a standard curve based on known concentrations of DNA standard dilutions. Next, four separate amplicon libraries (containing 1.00*10^9^ molecules/μl per sample) were prepared, representing two libraries for each primer pair used, including one for the samples taken in winter and one for the samples taken in summer. The quality of the resulting libraries was assessed using an Agilent Bioanalyzer 2100 with high‐sensitivity chip (Agilent Technologies, Waldbronn, Germany). Each amplicon library was sequenced on a separate 1/8th Pico Titer Plate (PTP) section using the Roche GS‐FLX+ instrument with Titanium chemistry according to manufacturer's instructions (Roche Applied Science, Mannheim, Germany).

Sequences obtained from the 454‐pyrosequencing run were assigned to the appropriate sample based on their barcodes and primer sequences, allowing zero discrepancies, and were subsequently trimmed using a custom Python script implemented within the USEARCH v. 7 analysis pipeline (Edgar, 2013). Sequences obtained from both PCR replicates per sample were combined and further trimmed based on a minimum Phred score of 30 (base call accuracy of 99.9%) averaged over a 50 bp moving window. Sequence length was determined for each primer pair assessed, with the average being 250 bp. Sequences with ambiguous base calls or homopolymers with a length of more than eight sequences were rejected, as were chimeric sequences detected by UCHIME 4.2 chimera detection (de novo algorithm) (Edgar, Haas, Clemente, Quince, & Knight, 2011). Remaining sequences were aligned and grouped into species‐level OTUs based on a 3% sequence dissimilarity cut‐off using the UPARSE algorithm implemented in USEARCH (Edgar, 2013). To minimize the risk of retaining sequences from sequencing errors, “global” singletons (i.e., OTUs representing only a single unique sequence in the entire dataset) were removed after UPARSE clustering (Brown et al., 2015; Waud, Busschaert, Ruyters, Jacquemyn, & Lievens, 2014). Due to uneven sequencing depth and correlation between number of sequence reads and number of OTUs per sample (data not shown), the number of sequences was rarefied to 1,000 sequences per sample for both bacteria and archaea. OTU representative sequences were assigned taxonomic identities using the “classify.seqs” command in Mothur (v. 1.36.1) (Schloss et al., 2009) against the Silva taxonomy database, v. Jul 2014 (Quast et al., 2013), manually curated to include organisms previously observed in activated sludge (Midas database; McIlroy et al., 2015).

Sequence data for all samples have been deposited in the Sequence Read Archive under the BioProject Accession PRJNA317527. OTU representative sequences were also submitted to GenBank under the Accession Numbers KX029477 to KX031991. A mock bacterial community DNA sample obtained from BEI Resources (HM‐276D; even, high‐concentration v5.1H) was included as a positive control for 454‐pyrosequencing using the bacterial primers, undergoing the same processing steps as all other samples. The results of this mock community were according to the expectations, illustrating the robustness of our results.

### 2.3 Real‐time quantitative PCR

To confirm and further assess the occurrence and distribution of two bacterial OTUs that could be specifically attributed to textile or municipal WWTPs (based on the 454 data), a qPCR analysis was performed. To this end, specific primers were designed for OTU217 (*Planctomyces* sp.) and OTU23 (*Rhodoferax* sp.), respectively (Table S2). Specificity of the primers was evaluated using the BLAST algorithm against GenBank, and further evaluated against the 454 datasets obtained in this study. Furthermore, qPCR analyses were performed for two bacterial genes involved in nitrogen removal, including the *amoA* and *nirK* gene, encoding a functional nitrifying (ammonium monooxygenase alpha subunit) and denitrifying enzyme (copper‐containing nitrite reductase) (for primers see Table S2) (Geets et al., 2007). Analyses were performed on an ABI StepOnePlus real‐time PCR system. Each reaction contained 1.0 μl 10× diluted genomic DNA, 0.5 μl of each primer (20 μM stock), 10.0 μl 2× iTaq universal SYBR Green supermix, and 8.0 μl nuclease‐free water. The qPCR run consisted of the same thermal profile as described above except for the annealing temperature which was 64°C (for OTU23 and OTU217) or 59° (for the *amoA* and *nirK* genes). At the end of each qPCR run, a melting curve analysis was performed as described above. Quantification was performed using a standard curve based on known concentrations of DNA standard dilutions from 10^7^ copies μl^−1^ down to 10^2^ copies μl^−1^. All qPCR analyses were conducted in duplicate.

### 2.4 Chemical analyses

In order to determine the environmental conditions to which the different microbial communities have been exposed, a number of chemical analyses were performed on the influent samples. Analyses were performed using Nanocolor test tubes and a Nanocolor 500D photometer (Macherey‐Nagel, Düren, Germany) according to manufacturer's instructions, and included measurement of ammonium (NH_4_^+^), nitrite (NO_2_^‐^), nitrate (NO_3_^‐^), COD, BOD, AOX, total phosphorus (TP), and total nitrogen (TN) concentrations. Conductivity (salinity) and pH were measured using the Inolab conductivity and pH level 2 benchtop meters, respectively (WTW, Weilheim, Germany) (for each test one measurement was performed). Additionally, dissolved oxygen (DO) and temperature were measured at the sampling site using a HI9146 DO and temperature meter, respectively (Hanna Instruments, Temse, Belgium).

### 2.5 Data analysis

Rarefaction curves were generated for each sample using the Vegan package (v. 2.2‐1) for R (Oksanen et al., 2013; R Development Core Team, 2013) to visualize the overall coverage of the studied microbial communities. Additionally, OTU richness, Chao1 and ACE richness estimators, and Shannon diversity were calculated using Mothur (v. 1.36.1) (Schloss et al., 2009). To detect OTUs that are specific for the textile or municipal activated sludge, we performed an indicator species analysis (ISA) in PC‐ORD 6 (Dufrêne & Legendre, 1997; McCune & Mefford, 2006). This analysis calculates an indicator value based on fidelity and relative abundance of an OTU in relation to the different sludge groups (textile or municipal). By definition, an indicator value of 100 (perfect indicator) implies that the presence of a given OTU identifies a treatment without error. The obtained indicator values were tested for significance using a Monte Carlo randomization test with 5,000 permutations.

For all chemical parameters, the mean and standard error were calculated for every sample origin (textile or municipal) and sampling time (February and July). Further on, chemical parameters were linked to origin and time using general linear models (univariate analysis) conducted in SPSS 22.0 for Windows (SPSS Inc., Chicago, IL). Nonmetric multidimensional scaling (NMDS) was performed on the sample * OTU matrix, using Bray‐Curtis distances to visualize differences in the microbial communities (Vegan package v. 2.2‐1). Subsequently, the measured chemical parameters were fitted onto the ordination and tested for significance based on a permutation test with 1000 iterations, using the function “envfit”. Finally, the fitted chemical parameters were compared to the results in the univariate analysis.

## 3 Results and Discussion

### 3.1 Archaeal and bacterial community composition

Bacterial and archaeal communities were profiled for a total of 22 activated sludge samples (11 samples from February and 11 from July) from five textile WWTPs, five municipal WWTPs and one plant dealing with both textile and municipal wastewater. Strikingly, low amounts of sequences were obtained for the archaeal communities in sludge of the textile WWTPs sampled in February (varying between five and 1,350 sequences; only one sample yielded more than 1,000 archaeal sequences (sample T2F). The reason for this is not clear, but can most likely be attributed to the 454 process as no differences in band intensities were observed across all samples studied when amplicons were loaded on an agarose gel (suggesting that the variability in the number of sequences cannot be attributed to the PCR step). As a result, only one out of the five samples taken in February from textile WWTPs was retained for further analysis of the archaeal community (sample T2F). For archaea, rarefaction curves generally tended to approach saturation; for bacteria, rarefaction curves did not reach clear saturation, indicating that further sequencing would be necessary to fully cover the bacterial diversity (Fig. S1). Based on Chao1, the sample coverage ranged between 56.9% and 100.0% for the archaeal communities and between 34.1 (outlier; in general >50%) and 92.8% for the bacterial communities (Table 1), suggesting that the most dominant community members were covered in our study. In total, 160 archaeal OTUs and 1645 bacterial OTUs were detected in the samples studied, global singletons excluded. Per sample, observed archaeal and bacterial richness varied between 18 and 60 OTUs (average of 39 ± 3.16 (SE)), and between 119 and 353 OTUs (average of 248 ± 14.5 (SE)), respectively (Table 1). Both archaeal (*p* = .01) and bacterial (*p* = 5.03E‐05) richness were significantly higher for samples from municipal WWTPs compared to those from textile WWTPs, indicating the presence of a specialized microbial community in activated sludge of textile WWTPs. More particularly, on an average, 29 (±2.94 (SE)) archaeal and 191 (±15.7 (SE)) bacterial OTUs were recovered from a textile WWTP sample, whereas on an average, 45 (±3.63 (SE)) and 298 (±12.3 (SE)) OTUs were recovered from a municipal WWTP sample, respectively. No significant differences were found between archaeal (*p* = .371) and bacterial (*p* = .622) richness for both seasons studied.

**Table 1:** Microbial community diversity indices for activated sludge from textile and municipal wastewater treatment plants (WWTPs)

Wastewater	WWTP	Sampling time	Sample	Archaea					Bacteria				
				Sobs^a^	Chao1	Coverage [%]^b^	Ace^c^	Shannon^d^	Sobs^a^	Chao1	Coverage [%]^b^	Ace^c^	Shannon^d^
Municipal	1	February	R1_F	48	51.93	92.43	56.05	1.98	233	336.21	69.30	420.79	4.71
Municipal	1	July	R1_J	60	85.50	70.18	75.22	2.66	353	672.05	52.53	1025.07	5.26
Municipal	2	February	R2_F	40	62.67	63.83	101.23	1.74	324	702.07	46.15	962.89	5.08
Municipal	2	July	R2_J	46	81.00	56.79	57.67	2.00	324	493.32	65.68	518.40	5.24
Municipal	3	February	R3_F	44	49.00	89.80	52.57	2.18	308	543.56	56.66	741.99	5.08
Municipal	3	July	R3_J	57	76.13	74.88	74.63	2.73	331	970.29	34.11	1267.17	5.05
Municipal	4	February	R4_F	40	49.17	81.36	49.77	1.50	274	438.41	62.50	446.92	4.87
Municipal	4	July	R4_J	44	70.25	62.63	71.10	2.18	260	359.92	72.24	393.20	4.86
Municipal	5	February	R5_F	18	23.00	78.26	23.23	1.07	314	555.38	56.54	957.48	4.93
Municipal	5	July	R5_J	50	68.20	73.31	61.99	2.72	258	340.73	75.72	372.89	4.88
Textile	1	February	T1_F	N.D.	N.D.	N.D.	N.D.	N.D.	233	322.02	72.36	392.87	4.89
Textile	1	July	T1_J	30	36.00	83.33	44.17	1.90	228	414.58	55.00	416.50	4.79
Textile	2	February	T2_F	21	24.75	84.85	26.37	0.96	181	270.52	66.91	264.25	4.01
Textile	2	July	T2_J	42	55.00	76.36	72.99	2.42	119	173.08	68.75	153.68	3.85
Textile	3	February	T3_F	N.D.	N.D.	N.D.	N.D.	N.D.	221	331.86	66.59	429.39	4.66
Textile	3	July	T3_J	29	44.00	65.91	32.24	1.96	141	151.90	92.82	192.31	4.10
Textile	4	February	T4_F	N.D.	N.D.	N.D.	N.D.	N.D.	174	245.03	71.01	259.15	4.21
Textile	4	July	T4_J	24	25.43	94.38	28.06	0.77	126	183.95	68.50	223.01	3.15
Textile	5	February	T5_F	N.D.	N.D.	N.D.	N.D.	N.D.	261	433.94	60.15	592.25	4.84
Textile	5	July	T5_J	30	39.00	76.92	56.58	1.96	222	291.45	76.17	305.62	4.79
Combined^e^	1	February	TR1_F	20	20.00	100.00	20.00	1.19	256	404.22	63.33	493.67	4.82
Combined^e^	1	July	TR1_J	58	79.38	73.07	77.13	2.27	306	441.71	69.28	510.93	4.98

N.D., not determined due to too few sequences.

^a^Observed richness.

^b^Observed richness/Chao1 estimate * 100.

^c^Abundance‐based coverage estimator.

^d^Shannon‐Wiener diversity index.

^e^Sample from a WWTP dealing with textile and municipal wastewater.

**Figure 1:**
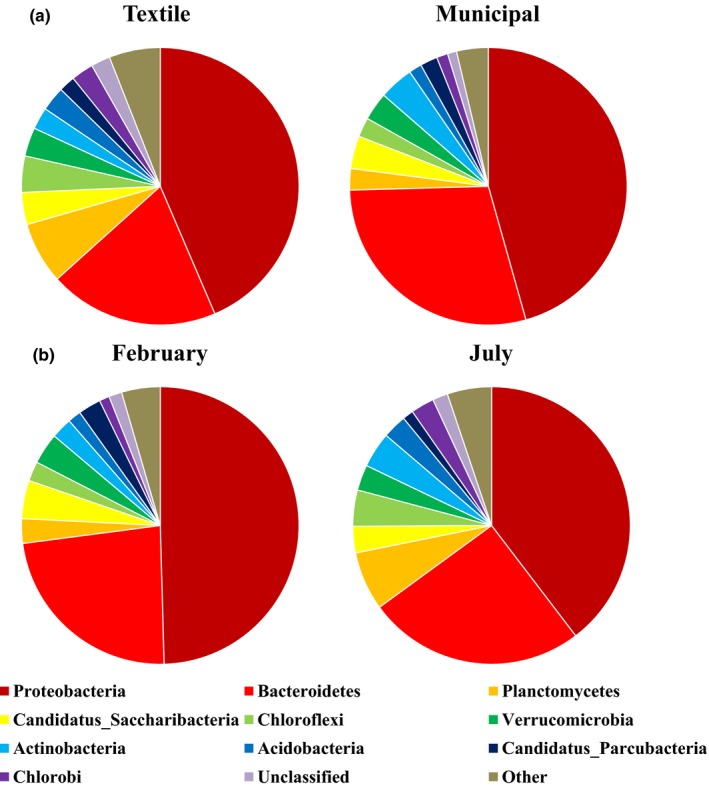
Relative abundance of bacterial phyla in activated sludge samples from textile and municipal wastewater treatment plants (WWTPs) (data combined for February and July; 22 samples) (a) sampled in February and July (data combined for textile and municipal WWTP samples; 22 samples) (b). Phyla representing less than 1% of the total amount of sequences are referred to as “Other”

Taxonomic assignment of the OTUs revealed the presence of two archaeal and 35 bacterial phyla in the activated sludge samples investigated in this study (Table S3). With regard to the archaea, Euryarchaeota represented 94.6% of the total number of sequences, while Thaumarchaeota represented 5.4%, respectively. This is in line with previous studies where Euryarchaeota were found to dominate the archaeal community in activated sludge from municipal WWTPs (Ju et al., 2014; Zhang et al., 2014). Further, as also noticed in previous studies on microbial communities in activated sludge (Hu et al., 2012; Saunders et al., 2016; Wang et al., 2012; Wei et al., 2015; Yang et al., 2014; Zhang et al., 2012; Zhao et al., 2014), Proteobacteria was the most abundant bacterial phylum detected (44.3% of the total number of sequences), followed by Bacteroidetes (24.8%). When comparing samples from municipal WWTPs with those from textile WWTPs, differences in relative abundance were observed for the archaeal phyla Euryarchaeota and Thaumarchaeota, being less and more abundant in samples from textile WWTPs, respectively. For bacteria, Bacteroidetes and Actinobacteria were more abundant in samples from municipal WWTPs compared to samples from textile WWTPs, while Planctomycetes, Chloroflexi, Acidobacteria, and Chlorobi were more abundant in samples from textile WWTPs (Fig. 1). When comparing samples from February with those from July, relative abundance increased by 9.15% for the archaeal phylum Thaumarchaeota with time at the cost of Euryarchaeota. For bacteria, relative abundance of Proteobacteria, Candidatus Saccharibacteria and Candidatus Parcubacteria was higher in February, whereas Planctomycetes, Actinobacteria, Chloroflexi, Chlorobi, and Acidobacteria were more abundant in July (Fig. 1). Thaumarchaeota represents a ubiquitous, relatively recently described archaeal phylum (Brochier‐Armanet, Boussau, Gribaldo, & Forterre, 2008), constituting the chemolithoautotrophic ammonia‐oxidizers that play important roles in biogeochemical cycles, such as the nitrogen cycle and carbon cycle (Offre, Spang, & Schleper, 2013; Tourna et al., 2011), and can therefore be considered as an important player in activated sludge processes (Park, Wells, Bae, Criddle, & Francis, 2006). Planctomycetes, Chloroflexi, Chlorobi, and Acidobacteria are known to contain halotolerant species that are found in extreme, heavily polluted habitats such as coastal salt marshes and contaminated soils (Canfora et al., 2014; Kutovaya, Lebedeva, Tkhakakhova, Ivanova, & Andronov, 2015; Strous et al., 1999). They fulfill important roles in global carbon, nitrogen and/or sulfur cycles, degrading carbohydrates, hydrocarbons, and heavy pollutants (Baker, Lazar, Teske, & Dick, 2015; García‐Fraile, Benada, Cajthaml, Baldrian, & Lladó, 2016; Glöckner et al., 2003; Hiras, Wu, Eichorst, Simmons, & Singer, 2016; Hug et al., 2013), making them important constituents of activated sludge processes.

**Figure 2:**
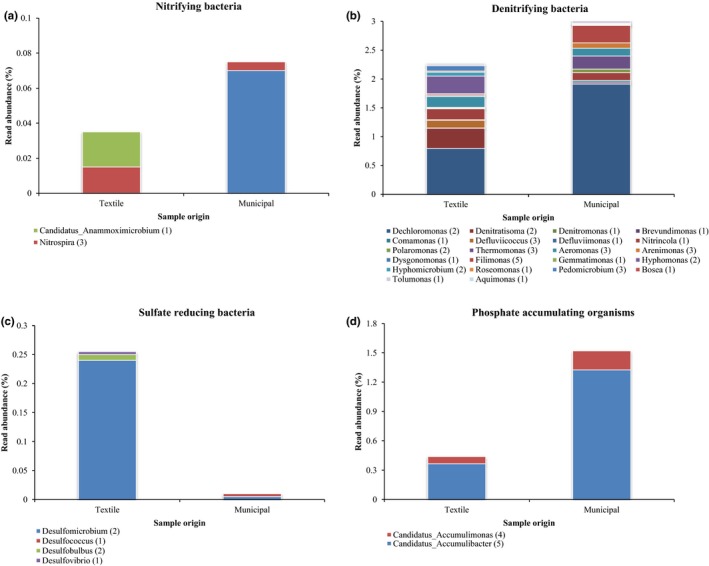
Read abundance of bacterial genera performing essential functions in activated sludge processes (nonexhaustive list), including nitrification (a), denitrification (b), sulfate reduction (c), and phosphate accumulation (d), in samples from textile and municipal wastewater treatment plants (WWTPs) (data combined for February and July; 22 samples). The number of Operational Taxonomic Units (OTUs) belonging to the genus is reported between brackets

In total, recovered species‐level OTUs could be classified in 21 archaeal and 259 bacterial genera (Table S4, approximately 40% of all OTUs), among which 76 genera were uniquely found in municipal WWTPs, 68 were uniquely found in textile WWTPs, and 136 were shared by both. A number of these genera have been functionally characterized and some of these are important to the successful operation of an activated sludge process, for example, through removal of carbon, ammonia, nitrate, sulfate, or phosphate or through their unwanted accumulating, bulking or foaming activity, which is typically caused by filamentous bacteria such as *Kouleothrix, Anaerolinea, Microthrix*, and *Thiothrix* (Nielsen, Kragelund, Seviour, & Nielsen, 2009; Rossetti, Tomei, Nielsen, & Tandoi, 2005). These filamentous bacteria were present (albeit at low densities) in both municipal (eight OTUs representing these four genera) and textile (four OTUs belonging to the genera *Anaerolinea*,* Microthrix*, and *Thiothrix*) WWTPs, but were generally more abundantly present in municipal WWTP samples (data not shown). Furthermore, nitrifying and denitrifying bacteria as well as phosphate‐accumulating bacteria showed a higher relative read abundance in municipal WWTPs (Fig. 2). This was confirmed by a qPCR analysis targeting the bacterial *amoA* and *nirK* genes, the first being involved in nitrification, the second in denitrification: samples from municipal WWTPs were significantly higher in *amoA* abundance during winter (*p* = 6.75E‐03) and summer (*p* = 8.34E‐05) as opposed to textile WWTP samples. Also, *nirK* abundance was higher in municipal samples, albeit not significantly (*p* = .518). These findings suggest that removal of ammonium, nitrate, and phosphate is likely more efficient in municipal WWTPs. Indeed, effluent measurements of the different municipal wastewaters showed an enhanced removal of phosphate and ammonium for the investigated municipal WWTPs in comparison with the investigated textile WWTPs (data not shown). In contrast, sulfate‐reducing bacteria were almost solely found in textile WWTPs (Fig. 2). Notably, a great number of OTUs (40) belonging to the genus *Planctomyces* (Planctomycetes) were specifically found in textile WWTPs, suggesting that these bacteria are well‐adapted to the conditions encountered in textile wastewater treatment systems, which, for example, are typically characterized by a high salt concentration. As such, these findings are in line with previous research showing that these bacteria can thrive in salt‐rich environments (Zhang et al., 2014). Additionally, members of the genera *Leucobacter* (Actinobacteria) and *Hydrogenophaga* (Proteobacteria) were predominantly found in the textile‐activated sludge samples. For both genera, species have been described isolated from dye wastewater (Kim & Lee, 2011; Yoon, Kang, Ryu, Jeon, & Oh, 2008). Additionally, some *Leucobacter* species have already been used in microbial consortia to degrade disperse and reactive dyes (Franciscon et al., 2010, 2015).

**Figure 3:**
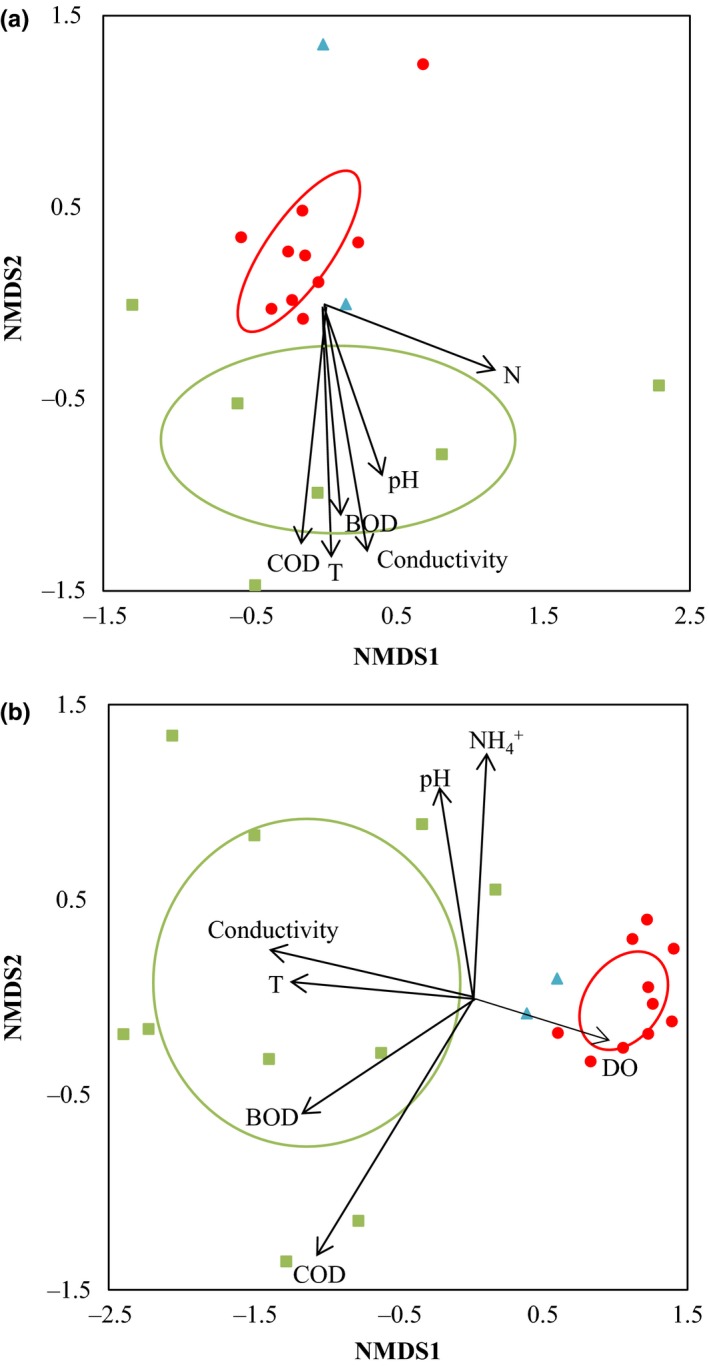
Nonmetric multidimensional scaling (NMDS) ordination (with environmental fit) of the archaeal (a; stress value = 0.153) and bacterial communities (b; stress value = 0.082) in activated sludge samples of textile (green squares) and municipal wastewater purification systems (red circles), as well as from one plant treating both municipal and textile wastewater (“combined”; blue triangles). Arrows represent environmental variables with significant correlations (Table 4). Length and orientation of the arrow is proportional to the direction and the amount of correlation between the ordination and the environmental variables

NMDS ordination of the community composition, inferred from the archaeal (*p* = .033; *R*² = 0.280) and bacterial (*p* = .001; *R*² = 0.663) OTU relative abundance, revealed that there was a significant difference (Goodness‐of‐Fit) between activated sludge samples from textile and municipal WWTPs (Fig. 3). Furthermore, samples from municipal WWTPs were much more similar than those from textile WWTPs (Fig. 3). Interestingly, NMDS ordination plotted the samples from the plant dealing with both municipal and textile wastewater (TR1) in between samples from municipal WWTPs on one hand and samples from textile WWTPs on the other hand (Fig. 3). Significant differences were found in community composition of archaeal communities sampled in February and July (*p* = .034; *R*² = 0.179), but not for bacteria (*p* = .694; *R*² = 0.014).

Strikingly, only one archaeal and one bacterial OTU was shared by all samples investigated. These included an OTU corresponding to *Methanosaeta* sp. (Euryarchaeota) and an OTU corresponding to an unidentified member of Proteobacteria, respectively. The first one covered approximately 23% of all archaeal sequences, while the second covered about 3% of all bacterial sequences. A core microbial community, consisting of five archaeal and 30 bacterial OTUs could be identified for municipal WWTPs that made up 33.0% and 19.3% of the total archaeal and bacterial sequences, respectively. Two of these bacterial genera, *Dechloromonas* and candidatus *Epiflobacter*, were also identified as one of the 23 to the genus‐level identified core genera in a recent study about activated sludge microbial communities in 13 Danish WWTPs (in total, 63 abundant genus‐level OTUs were identified based on a 6% 16S rRNA gene sequence dissimilarity cut‐off) (Saunders et al., 2016), suggesting a ubiquitous occurrence of these genera in municipal WWTPs. In contrast, only one archaeal and one bacterial OTU was shared by all textile WWTPs, suggesting that microbial communities in textile WWTPs are driven by diverse factors. ISA, allowing to identify one more given species/OTUs to serve as an indicator of a particular ecosystem, revealed the presence of two and six archaeal OTUs that could be attributed to textile and municipal WWTPs, respectively. For the bacteria, ISA revealed 10 and 34 indicator OTUs, respectively (Table S5). In order to confirm and generalize these results, all 22 samples investigated as well as six additional sludge samples from three textile WWTPs (sampled in February and July) and 10 additional samples from five municipal WWTPs (sampled in February and July) were subjected to qPCR analysis targeting two randomly selected indicator bacteria. These included OTU23, representing a member of the genus *Rhodoferax* (Proteobacteria), which was found as an indicator for municipal activated sludge, and OTU217, member of the genus *Planctomyces* (Planctomycetes), which was found as an indicator for activated sludge from textile WWTPs. OTU23 was found in all municipal WWTP samples analyzed, while it was absent in the textile WWTP samples. Additionally, OTU23 was found in activated sludge from the plant purifying both municipal and textile wastewater. OTU217 was found at five textile WWTPs, both in February and July (10 positive samples on a total of 16), while it was not detected in any sample from the municipal WWTPs (Table S6).

### 3.2 Environmental factors explaining differences in microbial communities

In order to determine environmental factors potentially explaining the differences in microbial communities in activated sludge from textile WWTPs and municipal WWTPs, several environmental variables were measured on the influent wastewater (Table 2). Samples from textile WWTPs had significantly higher salt levels and were higher in temperature (Table 2 and 3). Further, textile wastewater was found to contain a significantly higher organic load, as shown by the high COD and BOD values as opposed to municipal wastewater, whereas the DO level was significantly lower (Table 2 and 3), supporting previous findings (Verma et al., 2012). Also AOX values were slightly, but not significantly higher for textile wastewater. Little or no differences were found for NH_4_^+^, NO_2_^‐^, NO_3_^‐^, TP, TN, and pH (Table 2 and 3). Wastewater from the plant treating both municipal and textile wastewater was characterized by values situated between textile and municipal wastewater (Table 2). Notably, for some plants, differences were observed for particular influent characteristics (e.g., NH_4_^+^, TP, and COD) between the two sampling periods (Table 2), suggesting that these companies treat wastewaters with a variable composition.

**Table 2:** Influent wastewater characteristics

Wastewater	WWTP	Sampling time	Sample	Conductivity (mS/cm)	DO (ppm)	pH	Temperature (°C)	NH_4_^+^ (mg/L)	NO_2_^‐^ (mg/L)	NO_3_^‐^ (mg/L)	COD (mg O2/L)	BOD (mg O2/L)	TP (PO_4_‐P) (mg/L)	TN (mg/L)	AOX (mg/L)
Municipal	1	February	R1_F	0.959	6.27	6.97	10.4	29.1	0.02	1.3	167	6	2.22	26	0.03
Municipal	1	July	R1_J	1.000	1.81	7.52	19.9	43.7	0.02	3.1	199	25	6.66	38	0.03
Municipal	2	February	R2_F	0.580	10.96	7.01	8.3	8.8	0.02	1.3	21	2	0.73	9	0.04
Municipal	2	July	R2_J	1.000	0.04	7.64	20.6	53.0	0.06	2.1	240	22	7.30	53	0.03
Municipal	3	February	R3_F	0.652	10.27	6.88	9.4	0.2	0.02	24.4	52	2	1.65	10	0.04
Municipal	3	July	R3_J	1.000	1.05	7.59	9.6	80.0	0.02	3.2	380	34	8.68	75	0.09
Municipal	4	February	R4_F	1.080	9.02	6.77	11.0	17.8	0.04	5.2	519	116	6.35	33	0.04
Municipal	4	July	R4_J	2.000	0.53	7.76	10.3	41.2	0.03	1.3	92	13	14.78	33	0.04
Municipal	5	February	R5_F	1.541	8.52	7.08	14.2	41.0	0.02	1.6	307	29	2.97	43	0.04
Municipal	5	July	R5_J	2.000	3.09	7.62	21.1	61.0	0.02	2.4	261	34	5.33	52	0.05
Textile	1	February	T1_F	3.960	0.67	7.23	15.4	4.0	0.06	6.6	1476	90	1.75	16	0.18
Textile	1	July	T1_J	4.010	0.30	7.40	34.8	12.9	0.11	3.7	1724	300	9.84	24	0.06
Textile	2	February	T2_F	3.900	0.91	7.67	18.2	76.0	0.02	6.3	1153	125	4.19	96	0.14
Textile	2	July	T2_J	3.000	0.20	7.84	34.5	80.0	0.05	4.0	1124	320	9.34	91	0.14
Textile	3	February	T3_F	1.356	1.46	5.80	22.3	1.0	0.09	14.3	2818	28	2.66	51	0.08
Textile	3	July	T3_J	1.000	0.30	6.62	27.5	1.6	0.04	13.2	2993	480	5.61	42	0.04
Textile	4	February	T4_F	8.140	2.28	8.11	15.6	6.5	0.02	9.1	1426	134	14.20	29	0.02
Textile	4	July	T4_J	9.000	0.90	7.98	26.2	2.2	0.03	4.8	2771	130	7.42	7	0.44
Textile	5	February	T5_F	3.280	0.88	7.55	16.0	76.0	0.03	2.6	693	35	8.10	81	0.03
Textile	5	July	T5_J	2.000	0.42	7.53	25.2	37.1	0.06	8.8	786	62	7.79	54	0.02
Combined^a^	1	February	TR1_F	0.812	3.56	6.77	3.3	11.0	0.07	2.5	580	2	1.52	17	0.06
Combined^a^	1	July	TR1_J	1.000	0.99	7.67	10.3	47.5	0.09	5.4	720	43	9.79	53	0.06

AOX, Adsorbable organic halogens; BOD, biological oxygen demand; COD**,** chemical oxygen demand; DO**,** dissolved oxygen; TN, total nitrogen; TP, total phosphorus.

^a^Sample from a WWTP dealing with textile and municipal wastewater.

**Table 3:** Univariate analysis of the environmental variables corresponding to the analyzed activated sludge samples. Further, each environmental parameter is investigated through multiple comparisons in the origin subgroups (i.e., textile, municipal, and combined)

Environmental variable	Univariate analysis		Multiple comparisons (Tukey)		
	*F*‐value	*p*‐value	Textile/municipal	Textile/combined^a^	Combined^a^/municipal
Conductivity	6.309	.008	0.009	0.112	0.980
DO	5.150	.016	0.013	0.814	0.451
pH	0.097	.908	0.933	0.934	0.988
Temperature	9.641	.001	0.005	0.007	0.374
NH_4_^+^	0.196	.824	0.826	1.000	0.930
NO_2_^‐^	5.397	.014	0.071	0.311	0.025
NO_3_^‐^	0.720	.500	0.527	0.719	0.988
COD	15.177	.000	0.000	0.089	0.639
BOD	5.110	.017	0.018	0.189	0.977
AOX	1.694	.210	0.191	0.707	0.967
TP	0.336	.719	0.781	0.892	1.000
TN	0.601	.558	0.580	0.765	0.992

AOX, adsorbable organic halogens; BOD, biological oxygen demand; COD**,** chemical oxygen demand; DO**,** dissolved oxygen; TN**,** total nitrogen; TP, total phosphorus.

^a^Sample from a WWTP dealing with textile and municipal wastewater.

Archaeal community composition significantly (*p* < .05) varied with temperature, COD, BOD, conductivity, pH, and TN (Table 4), whereas the bacterial communities varied with temperature, conductivity, pH, DO, COD, BOD, and NH_4_^+^ (Table 4). When fitting the environmental variables on the NMDS ordination plot of the microbial communities (Fig. 3), temperature, conductivity, COD, BOD, and DO differentiated both archaeal and bacterial communities from textile and municipal WWTPs, with an increasing gradient toward samples from textile WWTPs except for DO, which shows an increasing gradient toward municipal activated sludge samples. For archaea, also TN and pH significantly divided samples from both groups. Fitting the environmental variables on the NMDS ordination of the bacterial communities also revealed that NH_4_^+^ and pH significantly discriminated samples from textile WWTPs, with an increasing gradient toward samples of two textile WWTPs (T2 and T5; irrespective of sampling time).

**Table 4:** Results of the permutation test of the nonmetric multidimensional scaling coordinates (NMDS 1 and NMDS 2) testing for significant relationships between activated sludge samples from textile and municipal wastewater treatment plants (WWTPs) and influent chemical variables

Archaea			Bacteria		
Environmental variable	*R*²	*p*‐value	Environmental variable	*R*²	*p*‐value
AOX	0.3795	.068	AOX	0.2964	.053
BOD	0.3892	.041*	BOD	0.4630	.007*
COD	0.4960	.014*	COD	0.8605	.001*
Conductivity	0.5433	.017*	Conductivity	0.5997	.001*
DO	0.2908	.088	DO	0.2868	.047*
NH_4_^+^	0.2985	.061	NH_4_^+^	0.4298	.006*
NO_2_^‐^	0.0759	.558	NO_2_^‐^	0.1211	.269
NO_3_^‐^	0.0201	.811	NO_3_^‐^	0.1149	.312
pH	0.3122	.046*	pH	0.3608	.012*
Temperature	0.5423	.005*	Temperature	0.4674	.004*
TN	0.3504	.034*	TN	0.2468	.066
TP	0.2796	.093	TP	0.1001	.343

AOX, adsorbable organic halogens; BOD, biological oxygen demand; COD**,** chemical oxygen demand; DO**,** dissolved oxygen; TN, total nitrogen; TP, total phosphorus.

The results are based on 999 permutations.

*R*² and *p*‐values are shown for the different environmental variables, where significant *p*‐values are indicated with *.

Altogether, our study shows that activated sludge from textile WWTPs harbors a highly specialized microbial community which is different from those from municipal WWTPs. High salinity, high organic loads, and a higher water temperature were important factors driving the microbial community composition in activated sludge from textile WWTPs. Earlier research confirms the importance of these factors in establishing microbial community structures (Griffiths, Ritz, Ebblewhite, & Dobson, 1998; Liu, Yang, Gong, & Su, 2008; Pietikäinen, Pettersson, & Bååth, 2005; Rietz & Haynes, 2003; Siggins, Enright, & O'Flaherty, 2011; Wakelin et al., 2012; Wang et al., 2012). In addition to these general parameters, other variables specifically linked to the textile dyeing industry (e.g., the dyes used, chemical additives etc.) are also likely to be involved in the mechanisms behind the assembly of these microbial communities. Further research is needed to unravel their importance in driving the assembly of textile WWTP communities. Future research building on our results could also aim at the identification of key players in the community that may be exploited for enhanced purification of textile wastewaters. In this regard, the phyla Planctomycetes (Bacteria) and Thaumarchaeota (Archaea), both abundantly present in activated sludge from textile WWTPs and possibly performing important functions in the purification of textile wastewaters, may provide promising candidates.

## Conflict of Interest

No conflict of interest declared.
