# Supplementary material for: Assessing the composition of microbial communities in textile wastewater treatment plants in comparison with municipal wastewater treatment plants
Source: Microbiologyopen. 2016 Sep 25;6(1):e00413. doi: 10.1002/mbo3.413 (PMC5300884; doi:10.1002/mbo3.413)
Supplement: Supplementary file 1 [file MBO3-6-0-s001.docx]

**Supporting information**

**Table S1** Fusion primer component sequences and structure^†^

| **454 GS-FLX+ Lib-L Adaptor Sequences** | | | | | | | | |
| --- | --- | --- | --- | --- | --- | --- | --- | --- |
| Application | | Adapter | | | Sequence (5’-3’) | | | |
| Forward Fusion Primers | | Adaptor “A” | | | CCATCTCATCCCTGCGTGTCTCCGACTCAG | | | |
| Reverse Fusion Primers | | Adapter “B” | | | CCTATCCCCTGTGTGCCTTGGCAGTCTCAG | | | |
| **Target Specific Primer Sequences** | | | | | | | | |
| Combination | | Target | Primer | | Sequence (5’-3’) | | | Direction |
| S-D-Bact-0341-b-S-17 | | V3-V4 | Bakt341F | | CCTACGGGNGGCWGCAG | | | Forward |
| S-D-Bact-0785-a-A-21 | |  | Bakt805R | | GACTACHVGGGTATCTAATCC | | | Reverse |
| S-D-Arch-0519-a-S-15 | | V4-V6 | A519F | | CAGCMGCCGCGGTAA | | | Forward |
| S-D-Arch-1041-a-A-18 | |  | Arch1017R | | GGCCATGCACCWCCTCTC | | | Reverse |
| **Multiplex Identifier Sequences (MIDs)**^‡^ | | | | | | | | |
| MID-1 | ACGAGTGCGT | | | MID-28 | | ACTACTATGT | | |
| MID-3 | AGACGCACTC | | | MID-30 | | AGACTATACT | | |
| MID-4 | AGCACTGTAG | | | MID-31 | | AGCGTCGTCT | | |
| MID-5 | ATCAGACACG | | | MID-32 | | AGTACGCTAT | | |
| MID-6 | ATATCGCGAG | | | MID-33 | | ATAGAGTACT | | |
| MID-8 | CTCGCGTGTC | | | MID-35 | | CAGTAGACGT | | |
| MID-17 | CGTCTAGTAC | | | MID-42 | | TCGATCACGT | | |
| MID-18 | TCTACGTAGC | | | MID-43 | | TCGCACTAGT | | |
| MID-19 | TGTACTACTC | | | MID-44 | | TCTAGCGACT | | |
| MID-20 | ACGACTACAG | | | MID-45 | | TCTATACTAT | | |
| MID-21 | CGTAGACTAG | | | MID-46 | | TGACGTATGT | | |
| **Multiplex Identifier (MID) Usage** | | | | | | | | |
| **Archaea** | | | | **Eubacteria** | | | | |
| Activated sludge T1 | | MID-1 | | Activated sludge T1 | | | MID-28 | |
| Activated sludge T2 | | MID-3 | | Activated sludge T2 | | | MID-30 | |
| Activated sludge T3 | | MID-4 | | Activated sludge T3 | | | MID-31 | |
| Activated sludge T4 | | MID-5 | | Activated sludge T4 | | | MID-32 | |
| Activated sludge T5 | | MID-8 | | Activated sludge T5 | | | MID-35 | |
| Activated sludge TR1 | | MID-6 | | Activated sludge TR1 | | | MID-33 | |
| Activated sludge R1 | | MID-17 | | Activated sludge R1 | | | MID-42 | |
| Activated sludge R2 | | MID-18 | | Activated sludge R2 | | | MID-43 | |
| Activated sludge R3 | | MID-19 | | Activated sludge R3 | | | MID-44 | |
| Activated sludge R4 | | MID-20 | | Activated sludge R4 | | | MID-45 | |
| Activated sludge R5 | | MID-21 | | Activated sludge R5 | | | MID-46 | |
| **Fusion Primer Concatemer Structure** | | | | | | | | |
| Forward Fusion Primers | | 5’ – Adapter “A” – MID – Forward Target Specific Primer – 3’ | | | | | | |
| Reverse Fusion Primers | | 5’ – Adapter “B” – Reverse Target Specific Primer – 3’ | | | | | | |
| ^†^Fusion Primers designed for Roche GS-FLX+ XLR70 instrument and Lib-L Titanium chemistry (Roche Applied Science, Mannheim, Germany). | | | | | | | | |
| ‡Multiplex identifier sequencers were selected from extended MID set TCB No: 005-2009 (Roche Applied Science, Mannheim, Germany). | | | | | | | | |

**Table S2** Real-time quantitative PCR primers used in this study.

| Target | Taxonomic affiliation (Silva v. 119/Midas) | Primer name | Primer sequence (5'-3') | Fragment length (bp) | Reference |
| --- | --- | --- | --- | --- | --- |
| OTU23 | *Rhodoferax* sp. | OTU23_F2 | GCA ATG CCG CGT GCA GGA C | 110 |  |
|  |  | OTU23_R1 | GGT ACC GTC ATT AGC CTC TC |  |  |
| OTU217 | *Planctomyces* sp. | OTU217_F1 | CCT GAC CGA GCG ACG CTA | 199 |  |
|  |  | OTU217_R1 | GAC CGC CTA CGC ACC CTG |  |  |
| *amoA* gene |  | amoA_1F | GGG GTT TCT ACT GGT GGT | 491 | Rotthauwe et al. 1997 |
|  |  | amoA_2R | CCC CTC KGS AAA GCC TTC TTC |  |  |
| *nirK* gene |  | nirK_1F | GGM ATG GTK CCS TGG CA | 514 | Braker et al. 1998 |
|  |  | nirK_5R | GCC TCG ATC AGR TTR TGG TT |  |  |

Rotthauwe, J. H., K.P Witzel, and W. Liesack. 1997. The ammonia monooxygenase structural gene *amoA* as a functional marker: molecular fine-scale

analysis of natural ammonia-oxidizing populations. Appl. Environ. Microbiol. 63:4704-4712.

Braker, G., A. Fesefeldt, and K.P.Witzel. 1998. Development of PCR primer systems for amplification of nitrite reductase genes (*nirK* and *nirS*) to detect

denitrifying bacteria in environmental samples. Appl. Environ. Microbiol. 64:3769-3775.

**Table S3** List of identified archaeal and bacterial phyla in the activated sludge samples from textile and municipal wastewater treatment systems (WWTPS) (Silva v.119/Midas).

| Archaea | | | | | | | | | |  |
| --- | --- | --- | --- | --- | --- | --- | --- | --- | --- | --- |
| Textile and municipal WWTPs |  | Additional in textile WWTPs | | | |  | Additional in municipal WWTPs | | |  |
| Euryarchaeota |  |  | | | |  |  | | |  |
| Thaumarchaeota |  |  | | | |  |  | | |  |
| Bacteria | | | | | | | | | |  |
| Textile and municipal WWTPs |  | Additional in textile WWTPs | | | |  | Additional in municipal WWTPs | | |  |
| Proteobacteria |  | Candidatus_OC31 | | | |  | Fusobacteria | | |  |
| Bacteroidetes |  | Armatimonadetes | | | |  | Fibrobacteres | | |  |
| Planctomycetes |  | Deferribacteres | | | |  | Synergistetes | | |  |
| Candidatus_Saccharibacteria |  | Candidatus_GOUTA4 | | | |  | Tenericutes | | |  |
| Chloroflexi |  | Candidatus_Microgenomates | | | |  | Candidatus_Dependentiae | | |  |
| Verrucomicrobia |  |  | | | |  |  | | |  |
| Actinobacteria |  |  | | | |  |  | | |  |
| Acidobacteria |  |  | | | |  |  | | |  |
| Candidatus_Parcubacteria |  |  | | | |  |  | | |  |
| Chlorobi |  |  | | | |  |  | | |  |
| Candidatus_Gracilibacteria | |  | |  |  | | | |  | |
| Firmicutes | |  |  | | | |  |  | |  |
| Candidatus_WCHB1-60 | |  |  | | | |  |  | |  |
| Cyanobacteria | |  |  | | | |  |  | |  |
| Candidatus_SHA-109 | |  |  | | | |  |  | |  |
| Lentisphaerae | |  |  | | | |  |  | |  |
| Gemmatimonadetes | |  |  | | | |  |  | |  |
| Candidatus_Absconditabacteria | |  |  | | | |  |  | |  |
| Spirochaetae | |  |  | | | |  |  | |  |
| Candidatus_BRC1 | |  |  | | | |  |  | |  |
| Chlamydiae | |  |  | | | |  |  | |  |
| Elusimicrobia | |  |  | | | |  |  | |  |
| Candidatus_Latescibacteria | |  |  | | | |  |  | |  |
| Nitrospirae | |  |  | | | |  |  | |  |
| Candidatus_TA06 | |  |  | | | |  |  | |  |
|  | |  |  | | | |  |  | |  |

**Table S4** List of identified archaeal and bacterial genera in the activated sludge samples from textile and municipal wastewater treatment systems (WWTPs) (Silva v.119/Midas).

| Archaea | | | | | | | |  |
| --- | --- | --- | --- | --- | --- | --- | --- | --- |
| Textile and municipal WWTPs |  | Additional in textile WWTPs | |  | | Additional in municipal WWTPs | |  |
| *Methanobacterium* |  | *Methanothermobacter* | |  | | *Candidatus_Methanomethylophilus* | |  |
| *Methanobrevibacter* |  | *Rice_Cluster_I* | |  | | *Methanimicrococcus* | |  |
| *Methanocorpusculum* |  | *Candidatus_Nitrosoarchaeum* | |  | | *Methanogenium* | |  |
| *Methanoculleus* |  | *Methanocalculus* | |  | |  | |  |
| *Methanofollis* |  |  | |  | |  | |  |
| *Methanolinea* |  |  | |  | |  | |  |
| *Methanomassiliicoccus* |  |  | |  | |  | |  |
| *Methanomethylovorans* |  |  | |  | |  | |  |
| *Methanoregula* |  |  | |  | |  | |  |
| *Methanosaeta* |  |  | |  | |  | |  |
| *Methanosarcina* |  |  | |  | |  | |  |
| *Methanosphaera* |  |  | |  | |  | |  |
| *Methanosphaerula* |  |  | |  | |  | |  |
| *Methanospirillum* |  |  | |  | |  | |  |
| Bacteria | | | | | | | |  |
| Textile and municipal WWTPs | |  | Additional in textile WWTPs | |  | | Additional in municipal WWTPs | |
| *188up* | |  | *Acetobacterium* | |  | | *12up* | |
| *32C6* | |  | *AKYG587* | |  | | *Akkermansia* | |
| *35-7A* | |  | *Alishewanella* | |  | | *Alistipes* | |
| *A0837* | |  | *Alterococcus* | |  | | *Aquabacterium* | |
| *A21b* | |  | *Aquicella* | |  | | *Aquaspirillum* | |
| *Acidovorax* | |  | *Azovibrio* | |  | | *Aquimonas* | |
| *Acinetobacter* | |  | *B45* | |  | | *Azoarcus* | |
| *Aeromicrobium* | |  | *Bauldia* | |  | | *Blastocatella* | |
| *Aeromonas* | |  | *Bosea* | |  | | *Brevifollis* | |
| *AK1DE1* | |  | *Bryobacter* | |  | | *Brevundimonas* | |
| *Altererythrobacter* | |  | *Bythopirellula* | |  | | *Bythopirellula* | |
| *Anaerolinea* | |  | *Caldithrix* | |  | | *Candidatus_Obscuribacter* | |
| *Arcobacter* | |  | *Candidatus_Alysiosphaera* | |  | | *CCM19a* | |
| *Arenimonas* | |  | *Candidatus_Anammoximicrobium* | |  | | *CD04* | |
| *B3-65* | |  | *Candidatus_Rhabdochlamydia* | |  | | *Cellvibrio* | |
| *B63* | |  | *Chthoniobacter* | |  | | *Chitinivorax* | |
| *Bacteriovorax* | |  | *CL500-3* | |  | | *Chryseobacterium* | |
| *Bacteroides* | |  | *Coxiella* | |  | | *Citrobacter* | |
| *BD1-7_clade* | |  | *Defluviicoccus* | |  | | *Comamonas* | |
| *Bdellovibrio* | |  | *Defluviimonas* | |  | | *Coprococcus* | |
| *Bifidobacterium* | |  | *Denitratisoma* | |  | | *CPB_P15&M38* | |
| *C1-B045* | |  | *Denitromonas* | |  | | *CPB_S18* | |
| *Candidatus_Accumulibacter* | |  | *Desulfobulbus* | |  | | *Crocinitomix* | |
| *Candidatus_Accumulimonas* | |  | *Desulfomicrobium* | |  | | *Cytophaga* | |
| *Candidatus_Epiflobacter* | |  | *Desulfovibrio* | |  | | *Desulfococcus* | |
| *Candidatus_Microthrix* | |  | *Dysgonomonas* | |  | | *Dialister* | |
| *Candidatus_Sarcinathrix* | |  | *Escherichia-Shigella* | |  | | *Dietzia* | |
| *Candidatus_Xenovorus* | |  | *Hyphomonas* | |  | | *Faecalibacterium* | |
| *Cloacibacterium* | |  | *K2-78* | |  | | *Fluviicola* | |
| *CPB_C22&F32* | |  | *Legionella* | |  | | *Fodinicola* | |
| *CPB_S60* | |  | *Leucobacter* | |  | | *Formivibrio* | |
| *CYCU-0281* | |  | *Levilinea* | |  | | *Geothrix* | |
| *Dechloromonas* | |  | *Lewinella* | |  | | *Haloferula* | |
| *Desulfomicrobium* | |  | *Litorilinea* | |  | | *Hydrotalea* | |
| *Dokdonella* | |  | *Mangroviflexus* | |  | | *Ilumatobacter* | |
| *Ferribacterium* | |  | *Marinicella* | |  | | *Jatrophihabitans* | |
| *Ferruginibacter* | |  | *Methylobacter* | |  | | *Kaga01* | |
| *Filimonas* | |  | *Methyloversatilis* | |  | | *Klebsiella* | |
| *Flavobacterium* | |  | *Methylovulum* | |  | | *Kouleothrix* | |
| *Fodinibacter* | |  | *MSB-1F2* | |  | | *Lacibacter* | |
| *Gemmata* | |  | *Nitrincola* | |  | | *Lactobacillus* | |
| *Gemmatimonas* | |  | *Parvibaculum* | |  | | *Lactococcus* | |
| *H106* | |  | *Pedomicrobium* | |  | | *Leadbetterella* | |
| *Haliangium* | |  | *Pelagibius* | |  | | *Leptospira* | |
| *Haliea* | |  | *Phycisphaera* | |  | | *LF_BF07* | |
| *Halioglobus* | |  | *Propionibacterium* | |  | | *MK04* | |
| *Haliscomenobacter* | |  | *Prosthecomicrobium* | |  | | *mle1-27* | |
| *Hirschia* | |  | *Proteiniphilum* | |  | | *Nitrosomonas* | |
| *HTG5* | |  | *Pseudolabrys* | |  | | *Nocardioides* | |
| *Hydrogenophaga* | |  | *Pseudonocardia* | |  | | *OM27_clade* | |
| *Hyphomicrobium* | |  | *Pseudospirillum* | |  | | *p-55-a5* | |
| *Iamia* | |  | *Reichenbachiella* | |  | | *Parabacteroides* | |
| *Ignavibacterium* | |  | *Rhodococcus* | |  | | *Pedobacter* | |
| *K2-30-37* | |  | *Rhodopirellula* | |  | | *Propioniciclava* | |
| *Lautropia* | |  | *Robiginitalea* | |  | | *Raoultella* | |
| *M05-Pitesti* | |  | *Roseimicrobium* | |  | | *Rhizobacter* | |
| *Macellibacteroides* | |  | *Staphylococcus* | |  | | *Runella* | |
| *Methylorosula* | |  | *Steroidobacter* | |  | | *Sandarakinorhabdus* | |
| *Microbacterium* | |  | *Sva0081_sediment_group* | |  | | *SBR2113* | |
| *ML817J-10* | |  | *Tabrizicola* | |  | | *sbr-gs28* | |
| *mle1-48* | |  | *Tessaracoccus* | |  | | *Sediminibacterium* | |
| *MNG7* | |  | *Vibrio* | |  | | *Spirochaeta* | |
| *Mycobacterium* | |  |  | |  | | *Sterolibacterium* | |
| *Nannocystis* | |  |  | |  | | *Subdoligranulum* | |
| *Nitrospira* | |  |  | |  | | *Tetrasphaera* | |
| *oca15* | |  |  | |  | | *Tolumonas* | |
| *Opitutus* | |  |  | |  | | *Tsukamurella* | |
| *Ottowia* | |  |  | |  | | *vadinBC27_wastewater-sludge_group* | |
| *P2CN44* | |  |  | |  | | *WCHB1-50* | |
| *P58* | |  |  | |  | |  | |
| *Paludibacter* | |  |  | |  | |  | |
| *PeM15* | |  |  | |  | |  | |
| *Peredibacter* | |  |  | |  | |  | |
| *Perlucidibaca* | |  |  | |  | |  | |
| *Phaselicystis* | |  |  | |  | |  | |
| *PHOS-HE28* | |  |  | |  | |  | |
| *PHOS-HE31* | |  |  | |  | |  | |
| *Pir4_lineage* | |  |  | |  | |  | |
| *Pirellula* | |  |  | |  | |  | |
| *Piscinibacter* | |  |  | |  | |  | |
| *Planctomyces* | |  |  | |  | |  | |
| *Polaromonas* | |  |  | |  | |  | |
| *Prevotella* | |  |  | |  | |  | |
| *Prosthecobacter* | |  |  | |  | |  | |
| *Proteiniclasticum* | |  |  | |  | |  | |
| *Pseudomonas* | |  |  | |  | |  | |
| *QEDR3BF09* | |  |  | |  | |  | |
| *QEEB1BB10* | |  |  | |  | |  | |
| *Ramlibacter* | |  |  | |  | |  | |
| *Reyranella* | |  |  | |  | |  | |
| *Rhizomicrobium* | |  |  | |  | |  | |
| *Rhodobacter* | |  |  | |  | |  | |
| *Rhodoferax* | |  |  | |  | |  | |
| *Rickettsia* | |  |  | |  | |  | |
| *rJ14* | |  |  | |  | |  | |
| *Roseomonas* | |  |  | |  | |  | |
| *Sandaracinus* | |  |  | |  | |  | |
| *SBR1029* | |  |  | |  | |  | |
| *SBRFL126* | |  |  | |  | |  | |
| *S-Btb7_22* | |  |  | |  | |  | |
| *Schlesneria* | |  |  | |  | |  | |
| *Shewanella* | |  |  | |  | |  | |
| *Simplicispira* | |  |  | |  | |  | |
| *Skagen138* | |  |  | |  | |  | |
| *Skagenf80* | |  |  | |  | |  | |
| *SM1A02* | |  |  | |  | |  | |
| *Sorangium* | |  |  | |  | |  | |
| *spb280* | |  |  | |  | |  | |
| *Sphingobium* | |  |  | |  | |  | |
| *Sphingopyxis* | |  |  | |  | |  | |
| *Stella* | |  |  | |  | |  | |
| *Streptococcus* | |  |  | |  | |  | |
| *Sulfuricurvum* | |  |  | |  | |  | |
| *Sulfuritalea* | |  |  | |  | |  | |
| *Sulfurospirillum* | |  |  | |  | |  | |
| *Tepidicella* | |  |  | |  | |  | |
| *Thermomonas* | |  |  | |  | |  | |
| *Thiothrix* | |  |  | |  | |  | |
| *Trichococcus* | |  |  | |  | |  | |
| *Turneriella* | |  |  | |  | |  | |
| *Verrucomicrobium* | |  |  | |  | |  | |
| *Woodsholea* | |  |  | |  | |  | |
| *Zoogloea* | |  |  | |  | |  | |
| *Zymomonas* | |  |  | |  | |  | |

**Table S5** Results of indicator species analysis (p < 0.05).

| Archaea | | | | | | |
| --- | --- | --- | --- | --- | --- | --- |
| Municipal WWTP | | |  | Textile WWTP | | |
| Indicator OTU | Taxonomic affiliation (Silva v.119/Midas) | P-value |  | Indicator OTU | Taxonomic affiliation (Silva v.119/Midas) | P-value |
| OTU_1 | *Methanosaeta* sp. | 3.40E-03 |  | OTU_432 | Thaumarchaeota | 4.22E-02 |
| OTU_91 | *Methanospirillum* sp. | 3.06E-02 |  | OTU_58 | Euryarchaeota | 4.94E-02 |
| OTU_309 | *Methanosaeta* sp. | 7.40E-03 |  |  |  |  |
| OTU_29 | *Methanospirillum* sp. | 1.86E-02 |  |  |  |  |
| OTU_172 | *Methanosaeta* sp. | 3.56E-02 |  |  |  |  |
| OTU_97 | Euryarchaeota | 3.94E-02 |  |  |  |  |
| Bacteria | | | | | | |
| Municipal WWTP | | |  | Textile WWTP | | |
| Indicator OTU | Taxonomic affiliation (Silva v.119/Midas) | P-value |  | Indicator OTU | Taxonomic affiliation (Silva v.119/Midas) | P-value |
| OTU_4 | *Zoogloea* sp. | 2.00E-04 |  | OTU_296 | *Hyphomicrobium* sp*.* | 4.00E-04 |
| OTU_47 | Actinobacteria | 2.00E-04 |  | OTU_200 | CPB_S60 (Proteobacteria) | 1.90E-02 |
| OTU_97 | Proteobacteria | 4.00E-04 |  | OTU_430 | *Hydrogenophaga* sp. | 2.66E-02 |
| OTU_120 | Bacteroidetes | 2.00E-04 |  | OTU_212 | Proteobacteria | 2.82E-02 |
| OTU_141 | Bacteroidetes | 2.00E-04 |  | OTU_595 | Proteobacteria | 2.68E-02 |
| OTU_262 | *Aquabacterium* sp. | 2.00E-04 |  | OTU_33 | Proteobacteria | 3.22E-02 |
| OTU_273 | *Zoogloea* sp. | 4.00E-04 |  | OTU_219 | Cyanobacteria | 3.46E-02 |
| OTU_444 | Bacteroidetes | 2.00E-04 |  | OTU_940 | ML817J-10 (Actinobacteria) | 3.34E-02 |
| OTU_126 | Bacteroidetes | 2.00E-04 |  | OTU_791 | mle I-48 (Chloroflexi) | 2.96E-02 |
| OTU_42 | Proteobacteria | 4.00E-03 |  | OTU_135 | Verrucomicrobia | 1.80E-02 |
| OTU_217 | *Planctomyces* sp. | 4.00E-03 |  |  |  |  |
| OTU_175 | Bacteroidetes | 4.40E-03 |  |  |  |  |
| OTU_512 | *Dechloromonas* sp. | 5.00E-03 |  |  |  |  |
| OTU_684 | Bacteroidetes | 1.40E-03 |  |  |  |  |
| OTU_99 | *Tepidicella* sp. | 9.60E-03 |  |  |  |  |
| OTU_23 | *Rhodoferax* sp. | 1.50E-02 |  |  |  |  |
| OTU_113 | *Rhodoferax* sp. | 2.50E-02 |  |  |  |  |
| OTU_17 | *Flavobacterium* sp. | 2.18E-02 |  |  |  |  |
| OTU_122 | *Ferruginibacter* sp. | 2.28E-02 |  |  |  |  |
| OTU_211 | *Brevifollis* sp. | 2.90E-02 |  |  |  |  |
| OTU_286 | Proteobacteria | 2.92E-02 |  |  |  |  |
| OTU_56 | Candidatus *Microthrix* | 3.30E-02 |  |  |  |  |
| OTU_261 | Proteobacteria | 2.98E-02 |  |  |  |  |
| OTU_147 | Proteobacteria | 3.38E-02 |  |  |  |  |
| OTU_133 | *Sulfuritalea* sp. | 3.64E-02 |  |  |  |  |
| OTU_636 | *Flavobacterium* sp. | 3.34E-02 |  |  |  |  |
| OTU_1 | *Ferribacterium* sp. | 3.68E-02 |  |  |  |  |
| OTU_86 | *Flavobacterium* sp. | 3.10E-02 |  |  |  |  |
| OTU_338 | *Turneriella* sp. | 3.10E-02 |  |  |  |  |
| OTU_109 | *Thermomonas* sp. | 4.44E-02 |  |  |  |  |
| OTU_269 | PHOS-HE28 (Bacteroidetes) | 3.78E-02 |  |  |  |  |
| OTU_558 | Candidatus *Accumulibacter* | 4.72E-02 |  |  |  |  |
| OTU_76 | Bacteroidetes | 3.78E-02 |  |  |  |  |
| OTU_283 | Candidatus BRC1 | 4.72E-02 |  |  |  |  |
| OTU_352 | Candidatus Gracilibacteria | 3.84E-02 |  |  |  |  |

**Table S6**  Distribution of two bacterial indicator operational taxonomic units (OTUs; identified at a 3% sequence dissimilarity cut-off) over several activated sludge samples from textile and municipal wastewater treatment plants (WWTPs), as determined by qPCR analysis^†^. OTU23 (*Rhodoferax* sp.) was found as an indicator for municipal activated sludge whereas OTU217 (*Planctomyces* sp.) was determined as an indicator for activated sludge from textile WWTPs.

| Wastewater | WWTP | Sampling time | Sample | OTU23^†^ | OTU217^†^ |
| --- | --- | --- | --- | --- | --- |
| Municipal | 1 | February | R1_F | + | - |
| Municipal | 1 | July | R1_J | + | - |
| Municipal | 2 | February | R2_F | + | - |
| Municipal | 2 | July | R2_J | + | - |
| Municipal | 3 | February | R3_F | + | - |
| Municipal | 3 | July | R3_J | + | - |
| Municipal | 4 | February | R4_F | + | - |
| Municipal | 4 | July | R4_J | + | - |
| Municipal | 5 | February | R5_F | + | - |
| Municipal | 5 | July | R5_J | + | - |
| Municipal | 6 | February | R6_F | + | - |
| Municipal | 6 | July | R6_J | + | - |
| Municipal | 7 | February | R7_F | + | - |
| Municipal | 7 | July | R7_J | + | - |
| Municipal | 8 | February | R8_F | + | - |
| Municipal | 8 | July | R8_J | + | - |
| Municipal | 9 | February | R9_F | + | - |
| Municipal | 9 | July | R9_J | + | - |
| Municipal | 10 | February | R10_F | + | - |
| Municipal | 10 | July | R10_J | + | - |
| Textile | 1 | February | T1_F | - | + |
| Textile | 1 | July | T1_J | - | + |
| Textile | 2 | February | T2_F | - | - |
| Textile | 2 | July | T2_J | - | - |
| Textile | 3 | February | T3_F | - | + |
| Textile | 3 | July | T3_J | - | + |
| Textile | 4 | February | T4_F | - | + |
| Textile | 4 | July | T4_J | - | + |
| Textile | 5 | February | T5_F | - | - |
| Textile | 5 | July | T5_J | - | - |
| Textile | 6 | February | T6_F | - | - |
| Textile | 6 | July | T6_J | - | - |
| Textile | 7 | February | T7_F | - | + |
| Textile | 7 | July | T7_J | - | + |
| Textile | 8 | February | T8_F | - | + |
| Textile | 8 | July | T8_J | - | + |
| Combined^¶^ | 1 | February | TR1_F | - | - |
| Combined^¶^ | 1 | July | TR1_J | - | - |
| ^†^Ct < 28 is considered positive (+), > 28 negative (-)  ^¶^Sample from a WWTP dealing with textile and municipal wastewater | | | | | |


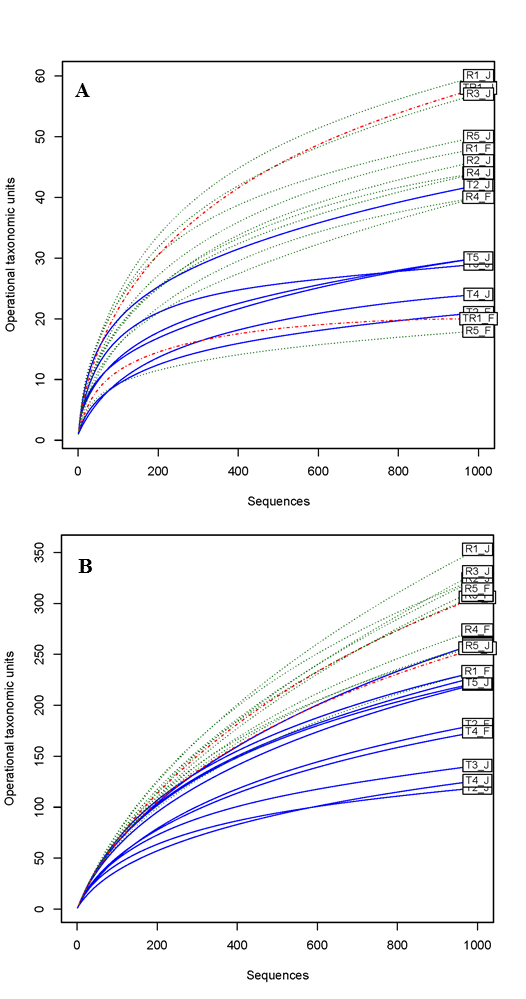


**Figure S1** Rarefaction curves generated for each activated sludge sample, illustrating accumulated number of operational taxonomic units (OTUs) (based on a DNA dissimilarity cut-off value of 3%) for archaea (A; 18 samples) and bacteria (B; 22 samples). For archaea, rarefaction curves generally tended to approach saturation; for bacteria rarefaction curves did not reach clear saturation. Solid blue lines represent activated sludge samples originating from textile WWTPs, green dotted lines represent samples from municipal WWTPs and red dot-dash lines are used for a plant dealing with both textile and municipal wastewater.


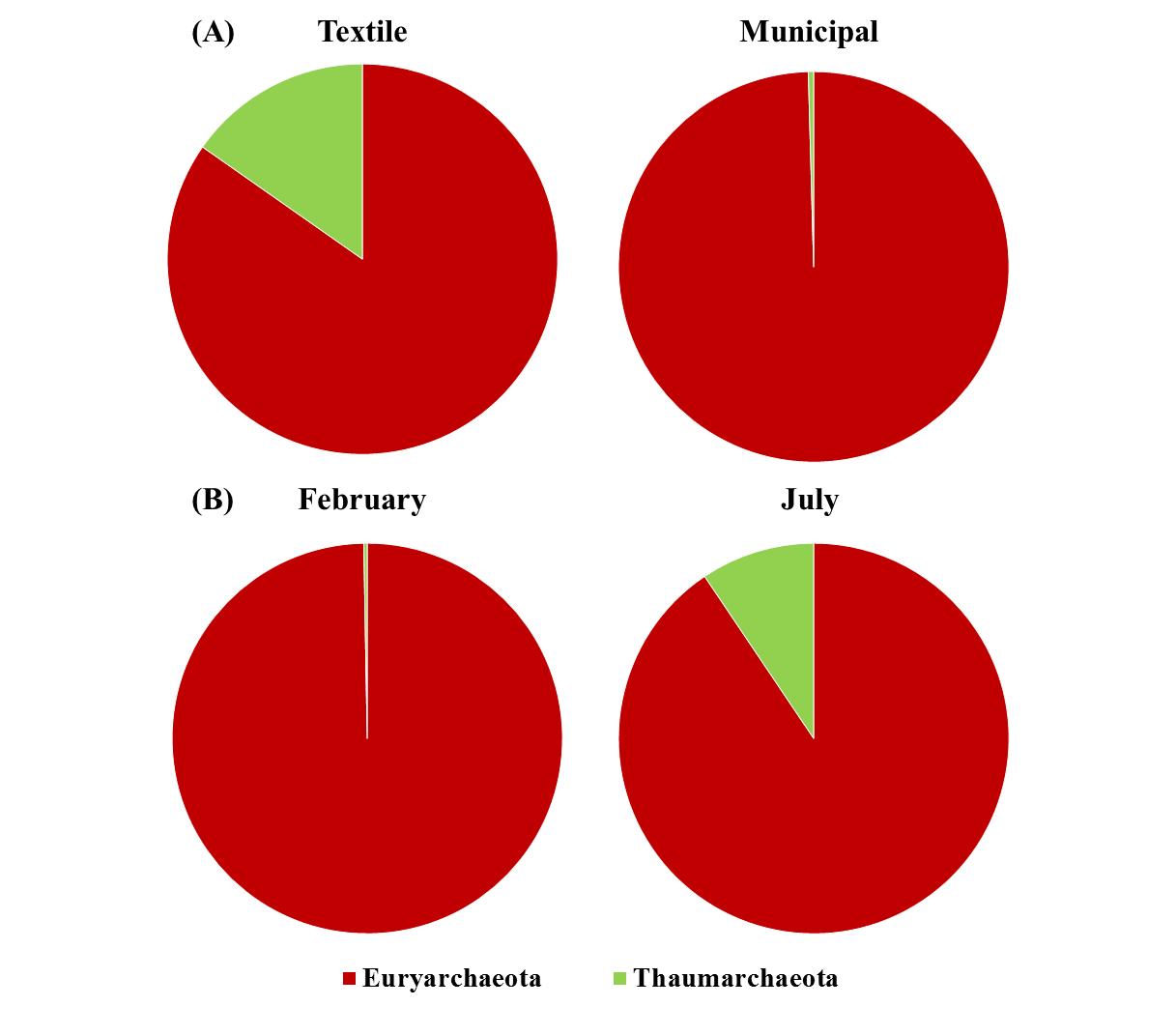
Figure S2 Archaeal relative phylum abundance in activated sludge samples from textile and municipal wastewater treatment plants (WWTPs) (data combined for February and July; 18 samples) (A) sampled in February and July (data combined for textile and municipal WWTP samples; 18 samples) (B).
